# Personal branding and personal marketing in the nursing job market: a scoping review

**DOI:** 10.1590/0034-7167-2024-0631

**Published:** 2026-07-27

**Authors:** Franciele Budziareck Neves, Laura Cavalcanti de Farias Brehmer, Bruna Nadaletti de Araújo, Gabriela Pereira Bozzetti, José Luís Guedes dos Santos, Mara Ambrosina de Oliveira Vargas

**Affiliations:** I Universidade Federal de Santa Catarina. Florianópolis, Santa Catarina, Brazil; II Diretoria de Vigilância Epidemiológica de Santa Catarina. Florianópolis, Santa Catarina, Brazil

**Keywords:** Job Market, Organization And Administration, Work, Nursing, Review., Mercado de Trabajo, Organización y Administración, Trabajo, Enfermería, Revisión.

## Abstract

**Objectives:**

to map, characterize, and synthesize existing evidence on personal branding and marketing strategies employed in the nursing job market, especially in relation to professional visibility, employability, and professional identity development.

**Methods:**

a scoping review, based on searches in databases and Google Scholar, using descriptors and terms related to “nursing”, “personal brand”, “personal branding”, and “personal marketing”.

**Results:**

of the 16,122 titles retrieved, 16 were included in the final sample. There is a variety in the use of concepts employed when relating to the placement of nursing professionals in the job market in the construction of their strategies to establish their personal brands.

**Conclusions:**

it is essential to advance the production of knowledge with well-defined conceptual objects and to deepen experiences regarding their employability. These advances strengthen professional identity, broaden visibility, and promote ethical practice in the nursing job market.

## RESUMEN

**Objetivos:**

mapear, caracterizar y sintetizar la evidencia existente sobre las estrategias de marca personal y marketing empleadas en el mercado laboral de enfermería, especialmente en relación con la visibilidad profesional, la empleabilidad y el desarrollo de la identidad profesional.

**Métodos:**

revisión del alcance, basada en búsquedas en bases de datos y Google Académico, utilizando descriptores y términos relacionados con “enfermería”, “marca personal”, “personal branding” y “marketing personal”.

**Resultados:**

de los 16.122 títulos recuperados, 16 se incluyeron en la muestra final. Se observa una variedad en el uso de conceptos empleados al abordar la inserción laboral de los profesionales de enfermería en la construcción de sus estrategias para consolidar su marca personal.

**Conclusiones:**

es fundamental impulsar la producción de conocimiento con objetos conceptuales bien definidos y profundizar en la experiencia relativa a su aplicabilidad. Estos avances fortalecen la identidad profesional, amplían la visibilidad y promueven la práctica ética en el mercado laboral de enfermería.

## INTRODUCTION

The healthcare field has been significantly influenced over the last few decades by ongoing global changes, which have directly impacted how healthcare services are delivered and managed. Nursing, as an essential area of training and professional practice in this scenario, is constantly called upon to adapt and evolve to meet nursing’s new demands, expanding its scope of action in a manner consistent with society’s growing needs, especially in relation to technological innovations. The job market, heavily influenced by political and economic factors, is unstable, subject to continuous changes, challenging working conditions, increasing competition, productivity demands, and visibility in different areas. From this reality emerged the need to define and adopt strategies that guarantee differentiation and sustainability of nursing professionals in their respective work settings^(1-3)^.

In this context, discussions emerged regarding professional identity construction and projection as a differentiation strategy. The concept of personal branding, understood as the strategic management of one’s personal brand, was initially proposed by Tom Peters (1997); since then, it has become part of debates on employability, reputation, and entrepreneurship. The concept of personal branding, which literally translates to personal brand management, emerged in 1997 when Tom Peters wrote an article entitled “The Brand Called You” for the American journal Fast Company, with the purpose of highlighting the idea that we are all a brand and that the strategic management of that brand has the potential to differentiate us professionally.

The central idea is that professionals not only possess a brand, but also have a duty to intentionally cultivate it, communicating attributes, values, and differentiators that generate trust and relevance^(4,5)^. Personal branding is how professionals are perceived and remembered by others. Therefore, effective management of this brand plays a crucial role in the evolutionary process of professional differentiation in the job market, since each individual, due to their uniqueness, presents distinct particularities and characteristics that make them one-of-a-kind^(6,7)^.

From a more specific perspective, Khedher^(4)^ explores personal branding as “a process of creating a unique personal identity, which develops an active communication of the brand identity, for a specific target market, and assessing its impact on reputation, in order to achieve personal and professional objectives”. By accepting the concept defended by Khedher^(4)^, it is possible to introduce personal marketing into the discussion, which is mistakenly understood as synonymous with personal branding. However, according to Santos^(8)^, personal marketing is a set of tools and strategies that, together, make professionals’ brand reach its target audience. In other words, personal marketing is not exactly the same as personal branding, but it is part of the process, assisting in the definition and execution of the brand’s best communication strategies with its audience.

Although personal branding and personal marketing have not traditionally been associated with nursing, in the current professional context, it is essential that professionals appropriate these concepts, if they wish, applying them as strategies in their daily lives. By investing in their own development and self-promotion techniques, professionals can expand their career opportunities and gain greater recognition for their professional skills, reflecting, among other things, in strengthening nursing’s identity. However, despite the professional benefits achieved by those who adopt personal branding and personal marketing strategies in their daily practice, these topics still present limitations when associated with nursing, which is explained by the frequent misuse of concepts associated with this strategy, such as “brand”, “branding”, “personal branding”, “personal marketing”, and “digital marketing”^(9)^.

Aiming to contribute to a deeper and more critical understanding of these concepts, this study poses the following research question: what is the scientific literature on personal branding and personal marketing strategies in the context of the nursing job market, especially in relation to professional visibility, employability, and professional identity development?

Finally, understanding and problematizing these dynamics has practical implications that extend beyond individual marketing. Promoting the development of reflective skills regarding professional identity can support professionals in positioning themselves ethically and strategically, negotiating fairer conditions and strengthening nursing’s social recognition as an essential and plural profession.

## OBJECTIVES

To map, characterize, and synthesize existing evidence on personal branding and marketing strategies employed in the nursing job market, especially in relation to professional visibility, employability, and professional identity development.

## METHODS

### Ethical aspects

Since this is a scoping review and used publicly available sources, this study does not require review by a Research Ethics Committee. However, ethical considerations in research involve database searches, data analysis, and discussion of results, and these must be rigorously followed.

### Study design

This study corresponds to an exploratory review of the scoping review type, based on the methodological structure according to JBI and Preferred Reporting Items for Systematic Reviews and Meta-Analyses extension for Scoping Reviews (PRISMA-ScR) guidelines^(10,11)^. Scoping reviews are recommended as a preliminary stage to a systematic review, aiming to examine emerging evidence, identify the types of evidence available in a specific field, analyze knowledge gaps, explore how research is conducted in a thematic area, and identify and elucidate the main characteristics or factors related to a concept or definition in the literature^(11)^.

### Methodological procedure

The review followed the five stages recommended by JBI: (1) formulating the research question; (2) identifying relevant studies; (3) selecting and including studies; (4) organizing the data; (5) compiling, synthesizing, and presenting the results (Peters et al., 2020). To ensure the integrity of this study and its methodological rigor, the PRISMA-ScR checklist was used in the review and writing process^(10)^.

The process of defining databases, choosing descriptors, and establishing a search protocol for studies in the databases was carried out by two independent professionals: a librarian and the principal investigator. This review was registered in the Open Science Framework, and the protocol is available at: DOI 10.17605/OSF.IO/Q546T.

To construct the research question, the PCC strategy was applied, which represents a mnemonic sequence relating to Population, Concept, and Context, defining: P - nursing; C - personal branding and personal marketing strategies; C - nursing job market (professional visibility, employability, and professional identity)^(11)^. Concerning study search and selection, the following guiding question was established: what is the scientific production on personal branding and personal marketing strategies in the context of the nursing job market, especially in relation to professional visibility, employability, and professional identity development?

### Data collection and organization

With the aim of identifying potentially relevant documents, the search was conducted in the following databases: National Library of Medicine (PubMed); Excerpta Medica database (EMBASE; Elsevier); Cumulative Index to Nursing and Allied Health Literature (CINAHL) (EBSCO); Scopus; Web of Science (Clarivate Analytics); *Literatura Latino-Americana e do Caribe em Ciências da Saúde* (LILACS - Virtual Health Library); *Bases de Dados da Enfermagem* (BDENF - Virtual Health Library); ProQuest Dissertations & Theses Global (PQDT Global); *Coordenação de Aperfeiçoamento de Pessoal de Nível Superior* (CAPES) Thesis and Dissertation Catalog; *Biblioteca Digital Brasileira de Teses e Dissertações* (BDTD); and Google Scholar. The search was conducted using descriptors and/or their synonyms.

The controlled terms used to compose the search strategies were defined from the Health Sciences Descriptors and Medical Subject Headings (MeSH), combined according to the specificities of each database and using the Boolean operators AND and OR as follows: Population (P): “*Enfermagem*” OR “*Enfermeiros*” OR “*Enfermeiras e Enfermeiros*” OR “Enfermeir*” OR “*Enfermeria*” OR “*Enfermeros*” OR “*Enfermeras y Enfermeros*” OR “enfermer*” OR “Nursing”[MeSH] OR “Nursing” OR “Nurs*” OR “Nurses”[MeSH]” OR “Nurses” AND; Concept (C): “Marca*” OR “Brand*” AND; Context (Co): “Mercadologi*” OR “*Mercadotecnía*” OR “Marketing”[MeSH] OR “Marketing”.

The searches were conducted between April and June 2023. During this period, all studies were accessed through the CAPES Journals Portal, accessed remotely via the *Comunidade Acadêmica Federada*’s Virtual Private Network, a resource funded by the *Universidade Federal de Santa Catarina*.

Eligibility criteria comprised indexed references in all languages, without time limits, available in full, in diverse formats encompassing original studies with quantitative, qualitative, mixed methods approaches, primary studies, systematic reviews, meta-analyses and/or meta-syntheses, as well as books, guidelines, consensus documents, editorials, blogs, and press releases.

An initial selection of studies was carried out by the researchers, based on reading the titles and abstracts. When there were doubts about the relevance of a study based on its abstract, the full text was selected for analysis. In addition to verifying whether the studies answered the guiding question of this review, the full text was examined to determine the final sample.

To operationalize the study selection process, the EndNote^®^ bibliographic reference management program was used. The program made it possible to locate duplicate references, create groups according to selection criteria (inclusion and exclusion), and build the review database.

To extract the data, a spreadsheet was created in Microsoft Excel^®^ containing the following elements: author/year; database; title; country/city; language; objective; study design; participants; context; conceptual approach of the object of study.

Results are presented descriptively, configuring, through charts, a mapping of the profile of studies that answer the research question. Through thematic analysis employed inductively, based on an exploratory and in-depth reading of the studies in the sample, predefined units of meaning were identified, such as personal marketing, personal branding, marketing, and branding, associated with, respectively, codes that formed the thematic categories, concepts, and their associated elements in the studies.

## RESULTS

**Figure 1:**
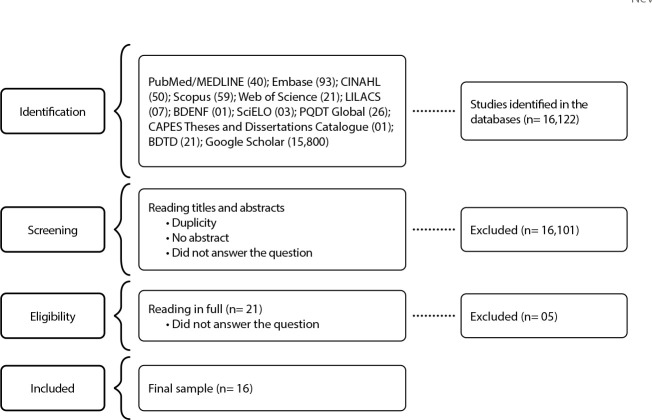
Flowchart for selecting studies for the scoping review, 2023

The initial search identified 16,122 titles; of these, 40 were in PubMed/MEDLINE; 93 were in EMBASE (Elsevier); 50 were in CINAHL (EBSCO); 59 were in Scopus (Elsevier); 21 were in Web of Science; seven were in LILACS; one in BDENF; three in SciELO; 26 in PQDT Global; one in the CAPES Thesis and Dissertation Catalog; 21 in BDTD; and 15,800 in Google Scholar. It should be noted that, in this last search engine, which is not specifically a database, the search strategy focused on identifying the combination of terms in the titles. After screening, only 21 were selected for full reading, based on an assessment of relevance using the title and abstract retrieved from the databases. Five other references were excluded from this process because they did not answer the guiding question. Thus, 16 titles comprised the sample for this review, according to Figure 1.

The studies in the sample were published between 2002 and 2023. Chart 1 shows the characteristics of studies in ascending chronological order.

**Chart 1:** Characterization of studies according to author/year, database, title, country, language, objective, document type/method, and context, Florianópolis, Santa Catarina, Brazil, 2023

Author(s)/year	Database	Title	Country	Language	Objective	Study design	Context
Pinkerton S. (2002)^(12)^	CINAHL	Marketing and Branding	United States of America	English	Discuss the advantages of marketing and branding for nurses, and describe specific examples of marketing and branding.	Reflection	General
Moura GMSS. (2003)^(13)^	Google Scholar	*Enfermagem e Marketing: uma introdução ao tema*	Brazil	Portuguese	Offer nurses an introductory overview of marketing.	Reflection	Management
Davidhizar R. (2007)^(14)^	EMBASE	Does Having a “Brand” Help You Lead Others?	United States of America	English	Explore the relationship between the concept of personal branding and leadership ability in the healthcare sector.	Reflection	Leadership/management
Maués DSO. (2007)^(15)^	CAPES	*O marketing pessoal do enfermeiro: uma contribuição para a gerência de enfermagem*	Brazil	Portuguese	Describe the use of personal marketing in the daily professional life of care managers and analyze, based on nurses’ statements, the personal marketing strategies in the job market that contribute to the appreciation and/or depreciation of nurses’ professional image.	Qualitative research	Management
Baldwin KA, Lyons RL, Issel LM. (2011)^(16)^	PubMed	Creating a Brand Image for Public Health Nursing	United States of America	English	Describe the process and results of developing a marketable image aimed at increasing the visibility and public awareness of public health nurses and their work.	Qualitative research	Public health
Bush C. (2012)^(17)^	CINAHL	Brand You in Twenty One Two!	United States of America	English	Provide oncology nursing professionals with tools to strengthen their personal brand.	Editorial	Oncology
Walker DK. (2012)^(18)^	CINAHL	Building Your Brand Establish Your Expertise to Stand Out From the Crowd	United States of America	English	Provide guidelines and strategies for nurses, developing and highlighting their personal brand.	Career guide	Oncology
Ratcliffe J. (2014)^(19)^	CINAHL	Managing and developing a personal brand: actions, attitude and appearance	United States of America	English	Provide a holistic approach to developing a personal brand, emphasizing the importance of aligning actions, attitudes, and appearance.	Branding matters	Aesthetics
O’Connell E. (2017)^(20)^	CINAHL	Creative Ways to Get Your Foot in the Door: A Step by Step LNC - Marketing Guide	United States of America	English	Provide strategies for Legal Nurse Consultants to consolidate their position in the job market.	Career guide	Consulting
Davis NE. (2017)^(21)^	CINAHL	What’s Your Nursing Brand Saying?	United States of America	English	Guide nurses on the importance of developing and managing their personal brand.	Reflection	General
Davis NE. (2017)^(22)^	CINAHL	Leveraging the use of branding for nurses in business	United States of America	English	Explore how nurses can use the concept of branding to strengthen their presence in the job market.	Reflection	Entrepreneurship
Molina BS, Santos DF, Draganov PB (2018)^(23)^	Google Scholar	*Subsídios para o marketing pessoal do enfermeiro*	Brazil	Portuguese	Point out the resources for developing personal marketing and its benefits for nurses.	Literature review	Management
Godsey JA, Hayes T, Schertzer C, Kallmeyer R. (2018)^(24)^	Web of Science	Development and testing of three unique scales measuring the brand image of nursing	United States of America	English	Develop empirically sound instruments that can measure nurses’ perceptions of their professional brand image.	Quantitative research	General
Godsey J, Perrot B, Hayes T. (2020)^(25)^	EMBASE	Can brand theory help re-position the brand image of nursing?	United States of America	English	Examine recent quantitative field research that describes nurses’ perceptions of their current versus desired brand position.	Literature review	General
Godsey JA, Houghton DM, Hayes T. (2020)^(26)^	EMBASE	Registered nurse perceptions of factors contributing to the inconsistent brand image of the nursing profession	United States of America	English	Describe the factors that influence the inconsistent brand image of nursing.	Qualitative research	General
Godsey JA, Hayes T. (2023)^(27)^	CINAHL	All Nurses Are Leaders: 5 Steps to Reconstruct the Professional Identity and Brand Image of Nursing	United States of America	English	Discuss the lack of a consistent brand message for nursing.	Reflection	General

*CINAHL - Cumulative Index to Nursing and Allied Health Literature; EMBASE - Excerpta Medica dataBASE; PubMed - National Library of Medicine.*

**Figure 2:**
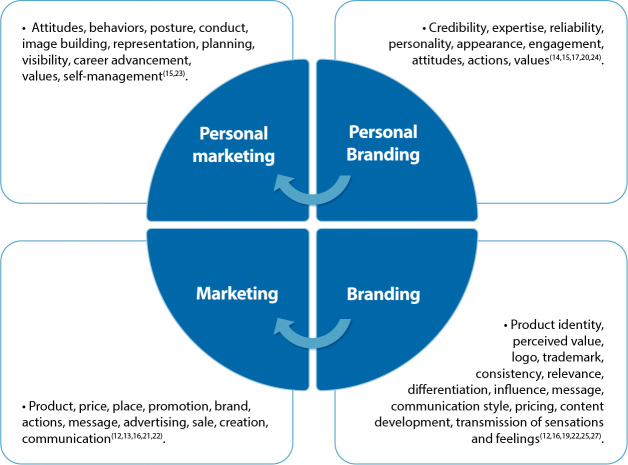
Conceptual approaches employed by sample studies for object treatment

An exploratory reading of the studies revealed a variation in the conceptual approaches employed to address the subject matter. The studies highlighted concepts of personal marketing and personal branding associated with professional profiles, as well as concepts of marketing and branding associated with products or services. The authors employed multiple terms in their respective studies, allowing for these associations, as shown in Figure 2.

## DISCUSSION

The mapping of studies showed that personal branding and personal marketing have been objects of interest in nursing since 2002. In the first decade, there are only four studies, and from 2011 to 2023, the others were identified. There is a predominance of North American studies^(13)^, indicating trends in the nursing job market in contexts considered to have high economic and social development, potentially favorable to the interests in exploring these objects. In relation to study design, only four are research papers, and six studies (the majority) are reflections.

The sample in this study does not stand out for specific research on personal branding and personal marketing in the nursing field. This highlights a gap in the production of knowledge that would provide a clearer and more consolidated conceptual and/or applied understanding of the subject. This finding is reinforced by the information presented in Chart 1, which shows the diversity of concepts used by the authors, confirming the lack of standardization in the language employed. However, this gap is not exclusive to the healthcare or nursing fields. Currently, there are many published studies on the subject in different areas of knowledge, in which a conceptual polysemy is also observed, without consistent efforts towards a more integrated understanding^(5)^.

This review mapped a conceptual rescue. In this way, it seeks the origin of the term “brand”, which comes from the English verb “burn”. Its use, as a synonym for brand, stems from the ancient practice of marking objects by burning them with hot iron, particularly used in branding cattle. This practice aimed to differentiate animals through their markings, enabling recognition among their peers. When we contextualize the concept within the corporate sphere, we can interpret it as the representation of a company’s brand. On the other hand, branding refers to the strategic process of building and managing corporate brands. This process involves the development of planned actions to promote the brand, aiming to make it more attractive, relevant, and positive in the audience’s and customers’ perception, which, in turn, increases its market value^(28)^.

In the meantime, personal branding refers to the strategic management of a personal brand. This process, while inheriting many practices from corporate branding, focuses on building an individual brand and promoting it in the market, aiming to add value and distinction to professional performance. Therefore, undoubtedly, managing a personal brand requires at least professional skill and strategy. Large, established brands recognized by different audiences in diverse contexts can teach lessons to people who want to stand out and thrive in the world of work^(29)^.

Related to this is marketing, which also differs in details from personal marketing, mainly in its focus and strategic approach. Marketing is a broad field encompassing all activities and processes used to promote and sell a company’s products or services. This includes market research, product development, pricing strategies, advertising campaigns, public relations, and distribution channels. The goal of marketing is to understand and meet consumer needs, creating value for both the company and the customer. Marketing strategies are aimed at promoting the company’s brand, increasing its visibility, attracting and retaining customers, and ultimately boosting sales of products or services and profitability^(30)^.

Personal marketing, on the other hand, is the application of marketing principles to promote a person’s brand. It involves defining and adopting assertive strategies to connect a personal brand with its target audience, in order to maximize its visibility and connection, leading to new opportunities, collaborations, career advancements, and professional goals. It presents itself as a tool in the contemporary landscape, where self-promotion as a differentiated professional who adds value to their field has become even more important^(8)^.

Based on the concepts above, it is possible to understand that branding, personal branding, and personal marketing, despite being distinct, can work in harmony to consolidate a robust and differentiated positioning, whether for a business or a professional. Each of these elements brings a different dimension to the construction of identity, recognition, and appreciation of a brand or professional. Therefore, branding and personal branding, together, form a solid foundation upon which marketing and personal marketing can operate, effectively communicating and promoting the unique identity to the target audience.

Of the 16 studies in this review, seven adopted the term “personal branding” to refer to professionals’ personal brand, demonstrating a diversity of terminology and possibly theoretical approaches in the nursing field^(14,15,17-20,24)^. This variation may indicate differences in academic traditions or in the cultural contexts in which the studies were conducted, as well as distinct interpretations of personal branding construction and management in nursing. Furthermore, the terminological choice suggests that the concept of personal branding is still under development, given that it is interpreted and applied in different ways by researchers, highlighting the richness and complexity of the topic, both for discussions and for its practical applicability.

It was also possible to identify that two studies used the expression “brand image” when addressing professionals’ personal brand^(26,27)^. It is important to highlight that, although “brand image” and “personal brand” are interconnected, they are not synonymous, since “brand image” refers to the general perception that others have of professionals, while “personal brand” involves the conscious construction of professional identity. According to Barbosa^(31)^, “brand image” is a multifaceted construction, supported by three integrated pillars: verbal and nonverbal communication, behaviors, and appearance. In the context of nursing, this means how professionals are perceived based on attributes such as vocabulary (verbal communication), gestures, and expressions (nonverbal communication) and professional performance.

Reflecting on this distinction is crucial to understanding how image and personal brand differ, yet can complement each other. “Brand image” is largely the result of a well-managed personal brand; it is how the audience perceives professionals based on the personal narrative they have built. However, personal branding is more proactive and comprehensive, including nursing professionals’ ongoing efforts to develop and communicate a cohesive and authentic professional identity. Above all, in scenarios of social inequality and precarious working conditions, branding could be recognized as a strategy grounded in public policies to provide visibility to professionals^(32,33)^.

The misuse of these terms can lead to a superficial understanding and ineffective strategies for advancing nursing in the job market, and may even promote inadequate understandings of professional identity. Therefore, it is essential that studies and practices in the nursing field recognize and respect these nuances, ensuring that the construction of a personal brand is approached holistically and intentionally. Furthermore, nine articles exclusively adopted the terms “brand” and “branding” to discuss personal branding in nursing, without mentioning the term “personal”^(12,16,19,22,25,27)^. This use of more general terms may indicate a tendency to apply corporate branding concepts directly to nursing professionals’ individual context. Although “brand” and “branding” are essential concepts for creating a recognizable identity, their direct application, without considering the specificities of personalization, can lead to a more impersonal and generic approach, potentially neglecting the need for authenticity and individuality, which is essential for personal branding^(29)^.

Of the seven studies that addressed marketing, five presented marketing from a broader perspective^(12,13,16,21,22)^, whereas two focused on the interface of personal marketing^(15,23)^. However, an analytical reflection reveals two important gaps. The first gap relates to the six articles that addressed marketing from a broader perspective, without highlighting the premises of personal marketing. This represents a significant challenge when adopted as a reference for building a personal brand for nursing professionals, because while traditional marketing may address generic strategies for promotion and visibility, personal marketing goes further, emphasizing individuals’ identity and values. Neglecting this personal dimension can result in an immediate, superficial, and ineffective approach that fails to capture the uniqueness and authenticity necessary to establish a recognizable professional reputation, directly impacting their positioning and connection with the target audience^(8)^. The second gap concerns the two studies that treated personal marketing in isolation, without integrating professional personal branding into the discussions. This fragmented approach ignores the complex interrelationship between personal marketing and personal branding, where the latter provides the essential foundation upon which personal marketing should be built. Without this connection, personal marketing strategies may become insufficient, focusing on aspects that do not contribute to a lasting, consistent, and sustainable professional reputation in nursing^(8)^.

From this perspective, recognizing the adopted concepts becomes fundamental for nursing to recognize its rightful place of importance in the job market. This self-(re)cognition can contribute, among other ways of identity formation, to the development of a relevant and sustainable professional identity in the long term. Furthermore, personalization facilitates the identification of specific opportunities and adaptation to different work contexts, maximizing the impact and effectiveness of personal branding and personal marketing strategies in a nursing career.

### Study limitations

Important limitations of this review include the absence of a cross-reference search strategy and the lack of criteria to assess the studies’ methodological quality. Furthermore, the limited number of included studies and the scarcity of research directly focused on nursing may have restricted the scope of the mapping. It is also possible that interpretative biases regarding the concepts influenced the study delimitation.

### Contributions to nursing

Provoking discussions about the (re)cognition of the concepts of branding, personal branding, and personal marketing in the job market, starting from professional training, represents filling a gap in nursing thinking and practice, fostering professional competencies that transcend the technical and clinical domain. By exploring these concepts in these professionals’ work, it is possible to contribute to overcoming challenges imposed by poor working conditions, highlighting nursing’s entrepreneurial and leadership character.

## FINAL CONSIDERATIONS

The results of this review indicated that, globally, there is still no clear and established conceptual framework on personal branding and personal marketing in nursing. The absence of a consolidated theoretical basis in this field points to the need for future studies that establish more cohesive, scientific definitions aligned with professionals and labor market context for nursing. These definitions should replace or complement the fragmented approaches currently available on personal branding and personal marketing in nursing. By proposing concepts more aligned with nursing contemporary demands, it is hoped to establish a theoretical and practical foundation that can guide professionals in promoting their personal brands and in deciding on the most appropriate personal marketing strategies. In this way, the aim is to promote and strengthen professional identity.

Therefore, it is crucial to emphasize that, although most of the studies examined have focused on branding and marketing, rather than personal branding and personal marketing when exploring personal branding in nursing, understanding these concepts is equally crucial for professionals to occupy the spaces they desire in the job market in various contexts.

## Data Availability

The research data are available only upon request.
