# Supplementary material for: Personal branding and personal marketing in the nursing job market: a scoping review
Source: Rev Bras Enferm. 2026 Jul 27;79:e20240631. doi: 10.1590/0034-7167-2024-0631 (PMC13411316; doi:10.1590/0034-7167-2024-0631)
Supplement: Translation

# Translation: Personal branding e marketing pessoal no mercado de trabalho da enfermagem: scoping review

**DOI:** 10.1590/0034-7167-2024-0631pt

**Authors:** Franciele Budziareck Neves, Laura Cavalcanti de Farias Brehmer, Bruna Nadaletti de Araújo, Gabriela Pereira Bozzetti, José Luís Guedes dos Santos, Mara Ambrosina de Oliveira Vargas

**Affiliations:** I Universidade Federal de Santa Catarina. Florianópolis, Santa Catarina, Brasil; II Diretoria de Vigilância Epidemiológica de Santa Catarina. Florianópolis, Santa Catarina, Brasil

**Keywords:** Mercado de Trabalho, Organização e Administração, Trabalho, Enfermagem, Revisão.

## Abstract

**Objetivos:**

mapear, caracterizar e sintetizar as evidências existentes sobre estratégias de personal *branding* e *marketing* pessoal empregadas no mercado de trabalho da enfermagem, especialmente em relação à visibilidade profissional, empregabilidade e desenvolvimento de identidade profissional.

**Métodos:**

*scoping review*, a partir de buscas em bases de dados e no Google Acadêmico, utilizando descritores e termos relacionados a “enfermagem”, “marca pessoal”, “*personal branding*” e “*marketing* pessoal”.

**Resultados:**

do total de 16.122 títulos recuperados, 16 foram incluídos na amostra final. Há uma variedade na utilização de conceitos empregados quando se relaciona à colocação dos profissionais de enfermagem no mercado de trabalho na construção de suas estratégias para se colocar em suas marcas pessoais.

**Conclusões:**

é essencial avançar na produção de conhecimentos com objetos bem delimitados conceitualmente e aprofundar experiências das suas empregabilidades. Esses avanços fortalecem a identidade profissional, ampliam a visibilidade e promovem a atuação ética no mercado de trabalho da enfermagem.

## INTRODUÇÃO

A área da saúde tem sido significativamente influenciada, ao longo das últimas décadas, pelas mudanças globais em curso, as quais têm impactado diretamente a forma como os serviços de saúde são prestados e gerenciados. A enfermagem, como área de formação e atuação profissional essencial desse cenário, é constantemente convidada a se adaptar e evoluir para atender às novas demandas da profissão, expandindo seu escopo de atuação, de modo coerente com as crescentes necessidades da sociedade, especialmente em relação às inovações tecnológicas. O mercado de trabalho, fortemente influenciado por fatores políticos e econômicos, principalmente, revela-se instável, estando sujeito a mudanças contínuas, condições laborais desafiadoras, concorrência crescente, exigências produtivistas e visibilidade em diferentes espaços. Dessa realidade, emergiu a necessidade da definição e adoção de estratégias que garantam a diferenciação e sustentabilidade dos profissionais de enfermagem nos seus respectivos cenários de atuação^(1-3)^.

Nesse cenário, emergiram discussões sobre a construção e projeção da identidade profissional como estratégia de diferenciação. O conceito de *personal branding*, entendido como a gestão estratégica da marca pessoal, foi inicialmente proposto por Tom Peters (1997); desde então, passou a integrar debates sobre empregabilidade, reputação e empreendedorismo. O conceito de *personal branding*, que, em sua tradução literal, significa gestão da marca pessoal, surgiu em 1997, quando Tom Peters escreveu um artigo intitulado “*The Brand Called You*” para a revista americana *Fast Company*, com o propósito de dar luz à ideia de que todos somos uma marca e a gestão estratégica da referida marca tem o potencial de nos diferenciar profissionalmente.

A ideia central é que o profissional não apenas possui uma marca, mas tem o dever de cultivá-la intencionalmente, comunicando atributos, valores e diferenciais que gerem confiança e relevância^(4,5)^. A marca pessoal é a maneira como os profissionais são percebidos e lembrados pelos outros. Assim, a eficaz gestão dessa marca desempenha um papel crucial no processo evolutivo na diferenciação profissional no mercado de trabalho, visto que cada indivíduo, por sua singularidade, apresenta particularidades e características distintas que o tornam único^(6,7)^.

Em uma ótica mais específica, Khedher^(4)^ explora o *personal branding* como “um processo de criação de uma identidade pessoal única, que desenvolve uma comunicação ativa da identidade de marca, para um mercado-alvo específico, e avaliando o seu impacto na reputação, de forma a atingir os objetivos pessoais e profissionais”. Ao aceitar o conceito defendido por Khedher^(4)^, é possível introduzir na discussão o *marketing* pessoal, que, equivocadamente, é compreendido como sinônimo do *personal branding*. Entretanto, segundo Santos^(8)^, o *marketing* pessoal é um conjunto de ferramentas e estratégias que, em conjunto, fazem com que a marca do profissional alcance o seu público-alvo. Em outras palavras, o *marketing* pessoal não é tal e qual o *personal branding*, mas faz parte do processo, auxiliando na definição e execução das melhores estratégias de comunicação da marca com o seu público.

Embora o *personal branding* e o *marketing* pessoal não tenham sido tradicionalmente associados à enfermagem, no contexto de atuação profissional atual, torna-se essencial que os profissionais se apropriem desses conceitos, se desejarem, aplicando-os como estratégias em seus cotidianos. Ao investir no seu próprio desenvolvimento e em técnicas de autopromoção, os profissionais podem ampliar suas oportunidades de carreira e obter maior reconhecimento por suas competências profissionais, refletindo, inclusive, no fortalecimento da identidade da profissão. Entretanto, apesar dos benefícios profissionais alcançados por aqueles que adotam as estratégias de *personal branding* e *marketing* pessoal em sua prática cotidiana, essas temáticas ainda apresentam limitações ao serem associadas à enfermagem, o que se explica pelo frequente uso equivocado dos conceitos associados a essa estratégia, tais como “*brand*”, “*branding*”, “*personal branding*”, “*marketing* pessoal” e “*marketing* digital”^(9)^.

Visando contribuir com a compreensão aprofundada e crítica desses conceitos, este estudo tem a seguinte pergunta de pesquisa: qual a produção científica sobre estratégias de *personal branding* (marca pessoal) e *marketing* pessoal no contexto do mercado de trabalho da enfermagem, especialmente em relação à visibilidade profissional, empregabilidade e desenvolvimento da identidade profissional?

Por fim, compreender e problematizar essas dinâmicas possui implicações práticas que extrapolam o *marketing* individual. Promover o desenvolvimento de competências reflexivas sobre identidade profissional pode apoiar os profissionais a se posicionar de forma ética e estratégica, negociando condições mais justas e fortalecendo o reconhecimento social da enfermagem como profissão essencial e plural.

## OBJETIVOS

Mapear, caracterizar e sintetizar as evidências existentes sobre estratégias de *personal branding* e *marketing* pessoal empregadas no mercado de trabalho da enfermagem, especialmente em relação à visibilidade profissional, empregabilidade e desenvolvimento de identidade profissional.

## MÉTODOS

### Aspectos éticos

Por se tratar de *scoping review* (revisão de escopo) e ter utilizado fontes de domínio público para sua realização, o presente estudo dispensa apreciação do Comitê de Ética em Pesquisa com Seres Humanos. Contudo, cuidados éticos em pesquisas envolvem buscas em bases de dados, análise de dados e discussão dos resultados, sendo rigorosamente seguidos.

### Tipo de estudo

Este estudo corresponde a revisão exploratória do tipo *scoping review*, fundamentada na estrutura metodológica, conforme JBI e nas diretrizes do *Preferred Reporting Items for Systematic Reviews and Meta-Analyses extension for Scoping Reviews* (PRISMA-ScR)^(10,11)^. A elaboração da scoping review é recomendada como uma etapa preliminar a uma revisão sistemática, visando examinar evidências emergentes, identificar os tipos de evidências disponíveis em um campo específico, analisar lacunas de conhecimento, explorar como a pesquisa é conduzida em uma área temática, e identificar e elucidar as principais características ou fatores relacionados a um conceito ou definição na literatura^(11)^.

### Procedimento metodológico

A revisão atendeu as cinco etapas preconizadas pelo JBI: (1) formulação da pergunta de pesquisa; (2) identificação de estudos pertinentes; (3) seleção e inclusão de estudos; (4) organização dos dados; (5) compilação, síntese e apresentação dos resultados (Peters *et al*., 2020). Para assegurar a integridade deste estudo e seu rigor metodológico, o *checklist* PRISMA-ScR foi utilizado na revisão e redação^(10)^.

O processo de definição de bases de dados, a escolha de descritores e o protocolo de busca dos estudos nas bases foram realizados por dois profissionais independentes: uma bibliotecária e a pesquisadora principal. Esta revisão foi registrada na *Open Science Framework*, e o protocolo está disponível em DOI 10.17605/OSF.IO/Q546T.

Para construção da pergunta da pesquisa, aplicou-se a estratégia PCC, que representa uma sequência mnemônica relativa à População, Conceito e Contexto, definindo: P - enfermagem; C - estratégias de *personal branding* (marca pessoal) e *marketing* pessoal; C - mercado de trabalho da enfermagem (visibilidade profissional, empregabilidade e identidade profissional)^(11)^. Com relação à busca e seleção dos estudos, estabeleceu-se a seguinte pergunta norteadora: qual a produção científica sobre estratégias de *personal branding* (marca pessoal) e *marketing* pessoal no contexto do mercado de trabalho da enfermagem, especialmente em relação à visibilidade profissional, empregabilidade e desenvolvimento da identidade profissional?

### Coleta e organização dos dados

Com o objetivo de identificar documentos potencialmente relevantes, a busca foi realizada nas seguintes bases de dados: *National Library of Medicine* (PubMed); *Excerpta Medica dataBASE* (EMBASE; Elsevier); *Cumulative Index to Nursing and Allied Health Literature* (CINAHL) (EBSCO); Scopus; Web of Science (*Clarivate Analytics*); Literatura Latino-Americana e do Caribe em Ciências da Saúde (LILACS - Biblioteca Virtual da Saúde); Bases de Dados da Enfermagem (BDENF - Biblioteca Virtual da Saúde); *ProQuest Dissertations & Theses Global* (PQDT Global); Catálogo de Teses e Dissertações da Coordenação de Aperfeiçoamento de Pessoal de Nível Superior (CAPES); Biblioteca Digital Brasileira de Teses e Dissertações (BDTD); e Google Acadêmico. A busca foi realizada por meio dos descritores e/ou seus sinônimos.

Os termos controlados para compor as estratégias de busca foram definidos a partir dos Descritores em Ciências da Saúde e dos *Medical Subject Headings* (MeSH), combinados conforme especificidades de cada base e utilizando-se os operadores booleanos *AND* e *OR* a seguir: População (P): “Enfermagem” *OR* “Enfermeiros” *OR* “Enfermeiras e Enfermeiros” *OR* “Enfermeir*” *OR* “Enfermeria” *OR* “Enfermeros” *OR* “Enfermeras y Enfermeros” *OR* “enfermer*” *OR* “*Nursing*”[MeSH] *OR* “*Nursing*” *OR* “Nurs*” *OR* “*Nurses*”[MeSH]” *OR* “*Nurses*” *AND*; Conceito (C): “Marca*” *OR* “Brand*” *AND*; Contexto (Co): “Mercadologi*” *OR* “Mercadotecnía” *OR* “*Marketing*”[MeSH] *OR* “*Marketing*”.

As buscas foram realizadas entre os meses de abril e junho de 2023. Nesse período, todas as publicações foram acessadas por meio do Portal de Periódicos da CAPES, acessado remotamente via *Virtual Private Network* da Comunidade Acadêmica Federada, recurso financiado pela Universidade Federal de Santa Catarina.

Os critérios de elegibilidade foram referências indexadas em todos os idiomas, sem limites temporais, disponíveis na íntegra, em formatos diversificados abrangendo estudos originais com abordagens quantitativa, qualitativa, métodos mistos, estudos primários, revisões sistemáticas, metanálises e/ou metassínteses, até livros, *guidelines*, consensos, editoriais, *blogs* e *releases*.

A seleção inicial dos estudos foi realizada pelas pesquisadoras, a partir da leitura dos títulos e resumos. Diante de dúvidas sobre a relevância de estudo baseado em seu resumo, a versão completa do texto foi selecionada para ser analisada. A leitura na íntegra possibilitou, além de verificar se os estudos respondiam à pergunta norteadora desta revisão, delinear a sua amostra final.

Para operacionalização do processo de seleção dos estudos, foi utilizado o programa de gerenciamento de referências bibliográficas *EndNote* ^®^. O programa possibilitou localizar as referências duplicadas, criar grupos de acordo com os critérios de seleção (inclusão e exclusão) e construir o banco de dados da revisão.

Para extração dos dados, foi elaborada uma planilha no *Microsoft Excel* ^®^ contendo os seguintes elementos: autor/ano; base de dados; título; país/cidade; idioma; objetivo; tipo de estudo, participantes; contexto; abordagem conceitual do objeto de estudo.

Os resultados são apresentados de modo descritivo, configurando, por meio de quadros, um mapeamento do perfil das publicações que respondem à pergunta de pesquisa. Por meio da análise temática empregada de forma indutiva, a partir da leitura exploratória e aprofundada dos estudos da amostra, foram identificadas unidades de significados pré-definidas, como *marketing* pessoal, *personal branding, marketing* e *branding*, associadas a, respectivamente, códigos que formaram as categorias temáticas, conceitos e seus elementos associados pelos estudos.

## RESULTADOS

A busca inicial identificou 16.122 títulos; desses, 40 foram no PubMed/MEDLINE; 93 foram na EMBASE (Elsevier); 50 foram na CINAHL (EBSCO); 59 foram na Scopus (Elsevier); 21 foram na Web of Science; sete foram na LILACS; um na BDENF; três na SciELO; 26 na PQDT Global; um no Catálogo de Teses e Dissertações da CAPES; 21 na BDTD; e 15.800 no Google Acadêmico. Cabe registrar que, neste último mecanismo de busca, que não se trata especificamente de uma base de dados, buscou-se nos títulos identificar a combinação dos termos na estratégia de busca. Após a triagem, apenas 21 foram selecionados para leitura na íntegra, conforme avaliação de pertinência a partir de título e resumo das recuperações em bases de dados. Deste processo, foram excluídas outras cinco referências, por não responderem à pergunta norteadora. Assim, 16 títulos compuseram a amostra desta revisão, de acordo com a Figura 1.

**Figura 1:** Fluxograma de amostra de seleção dos estudos para a *scoping review*, 2023

Os estudos da amostra foram publicados no período entre os anos de 2002 a 2023. O Quadro 1 apresenta, em ordem cronológica crescente, a caracterização das publicações.

**Quadro 1:** Caracterização dos estudos segundo autor/ano, base de dados, título, país, idioma, objetivo, tipo de documento/método e contexto, Florianópolis, Santa Catarina, Brasil, 2023

Autor(es)/ano	Base	Título	País	Idioma	Objetivo	Tipo de estudo	Contexto
Pinkerton S. (2002)^(12)^	CINAHL	*Marketing and Branding*	Estados Unidos da América	Inglês	Discutir as vantagens do *marketing* e da marca dos enfermeiros, e descrever exemplos específicos de *marketing* e *branding*.	Reflexão	Geral
Moura GMSS. (2003)^(13)^	Google Acadêmico	Enfermagem e *Marketing:* uma introdução ao tema	Brasil	Português	Oferecer aos enfermeiros uma visão introdutória sobre o *marketing*.	Reflexão	Gestão
Davidhizar R. (2007)^(14)^	EMBASE	*Does Having a ‘‘Brand’’ Help You Lead Others?*	Estados Unidos da América	Inglês	Explorar a relação entre o conceito de marca pessoal e a capacidade de liderança no setor de saúde.	Reflexão	Liderança/gestão
Maués DSO. (2007)^(15)^	CAPES	O *marketing* pessoal do enfermeiro: uma contribuição para a gerência de enfermagem	Brasil	Português	Descrever a utilização do *marketing* pessoal no cotidiano profissional do gerente do cuidado e analisar, segundo os depoimentos dos enfermeiros, as estratégias de *marketing* pessoal no mercado de trabalho que contribuem para valorização e/ou desvalorização da imagem profissional do enfermeiro.	Pesquisa qualitativa	Gestão
Baldwin KA, Lyons RL, Issel LM. (2011)^(16)^	PubMed	*Creating a Brand Image for Public Health Nursing*	Estados Unidos da América	Inglês	Descrever o processo e os resultados do desenvolvimento de uma imagem comercializável destinada a aumentar a visibilidade e a consciência pública dos enfermeiros de saúde pública e seu trabalho.	Pesquisa qualitativa	Saúde pública
Bush C. (2012)^(17)^	CINAHL	*Brand You in Twenty One Two!*	Estados Unidos da América	Inglês	Fornecer aos profissionais de enfermagem oncológica ferramentas para fortalecer sua marca pessoal.	Editorial	Oncologia
Walker DK. (2012)^(18)^	CINAHL	*Building Your Brand Establish Your Expertise to Stand Out From the Crowd*	Estados Unidos da América	Inglês	Fornecer diretrizes e estratégias para enfermeiros, desenvolvendo e destacando sua marca pessoal.	Guia de carreira	Oncologia
Ratcliffe J. (2014)^(19)^	CINAHL	*Managing and developing a personal brand: actions, attitude and appearance*	Estados Unidos da América	Inglês	Fornecer uma abordagem holística para o desenvolvimento de uma marca pessoal, enfatizando a importância de alinhar ações, atitudes e aparência.	*Branding matters*	Estética
O’Connell E. (2017)^(20)^	CINAHL	*Creative Ways to Get Your Foot in the Door: A Step by Step LNC - Marketing Guide*	Estados Unidos da América	Inglês	Fornecer estratégias para profissionais de enfermagem legal (*Legal Nurse Consultants*) para sua consolidação no mercado de trabalho.	Guia de carreira	Consultoria
Davis NE. (2017)^(21)^	CINAHL	*What’s Your Nursing Brand Saying?*	Estados Unidos da América	Inglês	Orientar os enfermeiros acerca da importância do desenvolvimento e gerenciamento da marca pessoal.	Reflexão	Geral
Davis NE. (2017)^(22)^	CINAHL	*Leveraging the* *use of branding for nurses in business*	Estados Unidos da América	Inglês	Explorar como os enfermeiros podem utilizar o conceito de *branding* para fortalecer sua presença no mercado de trabalho.	Reflexão	Empreendedorismo
Molina BS, Santos DF, Draganov PB (2018)^(23)^	Google Acadêmico	Subsídios para o marketing pessoal do enfermeiro	Brasil	Português	Apontar os subsídios para o desenvolvimento do *marketing* pessoal e seus benefícios para o enfermeiro.	Revisão de literatura	Gestão
Godsey JA, Hayes T, Schertzer C, Kallmeyer R. (2018)^(24)^	*Web of Science*	*Development and testing of three unique scales measuring the brand image of nursing*	Estados Unidos da América	Inglês	Desenvolver instrumentos empiricamente sólidos que pudessem medir as percepções dos enfermeiros sobre sua imagem de marca profissional.	Pesquisa quantitativa	Geral
Godsey J, Perrot B, Hayes T. (2020)^(25)^	EMBASE	*Can brand theory help re-position the brand image of nursing?*	Estados Unidos da América	Inglês	Examinar pesquisas de campo quantitativas recentes que descrevem as percepções da posição de marca atual versus desejada dos enfermeiros.	Revisão de literatura	Geral
Godsey JA, Houghton DM, Hayes T. (2020)^(26)^	EMBASE	*Registered nurse perceptions of factors contributing to the inconsistent brand image of the nursing profession*	Estados Unidos da América	Inglês	Descrever os fatores que influenciam a imagem de marca inconsistente da enfermagem.	Pesquisa qualitativa	Geral
Godsey JA, Hayes T. (2023)^(27)^	CINAHL	*All Nurses Are Leaders: 5 Steps to Reconstruct the Professional Identity and Brand Image of Nursing*	Estados Unidos da América	Inglês	Discutir a falta de mensagem de marca consistente da profissão de enfermagem.	Reflexão	Geral

*CINAHL - Cumulative Index to Nursing and Allied Health Literature; EMBASE - Excerpta Medica dataBASE; PubMed - National Library of Medicine.*

A leitura exploratória dos estudos desvelou uma variação entre as abordagens conceituais empregadas para o tratamento do objeto. Nos estudos, evidenciaram-se conceitos de *marketing* pessoal e *personal branding* associados ao perfil profissional, bem como conceitos de *marketing* e *branding* associados ao produto ou serviço. Observou-se o emprego, pelos autores em suas respectivas publicações, de múltiplos termos que permitiram estas associações conforme representado pela Figura 2.

**Figura 2:** Abordagens conceituais empregadas pelos estudos da amostra para o tratamento do objeto

## DISCUSSÃO

O mapeamento dos estudos evidenciou que o *personal branding* e o *marketing pessoal* são objetos de interesse da enfermagem desde 2002. Na primeira década, são apenas quatro estudos, e partir de 2011 até 2023, foram identificados os demais. Destaca-se predomínio de estudos norte-americanos^(13)^, indicando tendências do mercado de trabalho em enfermagem em contextos considerados de alto desenvolvimento econômico e social potencialmente favoráveis aos interesses em explorar os objetos. Em relação aos tipos de estudos, apenas quatro são pesquisas, e seis publicações (maioria) são reflexões.

A amostra deste estudo não se destaca por pesquisas específicas sobre *personal branding* e *marketing* pessoal na área da enfermagem. Evidencia-se, assim, uma lacuna na produção de conhecimentos que forneçam uma compreensão conceitual e/ou aplicada do objeto de forma mais clara e consolidada. Essa constatação é reforçada pelas informações apresentadas no Quadro 1, que mostram a diversidade de conceitos utilizados pelos autores, confirmando a ausência de padronização na linguagem empregada. Contudo, essa lacuna não é exclusiva da área da saúde ou da enfermagem. Atualmente, existem muitos estudos publicados sobre o tema em diferentes áreas do conhecimento, nas quais também se observa uma polissemia conceitual, sem esforços consistentes para uma compreensão mais integrada^(5)^.

Esta revisão mapeou um resgate conceitual. Dessa forma, busca-se a origem do termo “*brand*”, que provém do verbo “*burn*”, em inglês, cuja tradução significa “queimar”. O seu uso, enquanto sinônimo de marca, decorre da prática milenar de marcar objetos pela queima com ferro quente, particularmente utilizada na marcação do gado. Essa prática visava diferenciar os animais por meio de suas marcas, possibilitando o reconhecimento entre os seus semelhantes. Ao contextualizarmos o conceito no âmbito corporativo, podemos interpretá-lo como a representação da marca de uma empresa. Por outro lado, o *branding se* refere ao processo estratégico de construção e gestão de marcas corporativas. Esse processo envolve a elaboração de ações planejadas para promover a marca, visando torná-la mais atrativa, relevante e positiva na percepção do público e clientes, o que, por sua vez, aumenta o seu valor no mercado^(28)^.

Neste ínterim, o *personal branding* se refere ao gerenciamento estratégico da marca pessoal. Esse processo, embora herde muitas práticas do *branding* corporativo, foca na construção de uma marca individual e na sua promoção no mercado, visando agregar valor e distinção à atuação profissional. Logo, indubitavelmente, o gerenciamento de uma marca pessoal requer minimamente capacidade e estratégia profissional. As grandes marcas consolidadas e reconhecidas por diferentes públicos em contextos diversos podem ensinar lições às pessoas que desejam se destacar e prosperar no mundo do trabalho^(29)^.

Vinculado a isso, surge o *marketing,* o qual também se difere em detalhes do *marketing* pessoal, principalmente no objeto de foco e na abordagem estratégica. O *marketing* é um campo amplo que abrange todas as atividades e processos utilizados para promover e vender produtos ou serviços de uma empresa. Isso inclui pesquisa de mercado, desenvolvimento de produtos, estratégias de preços, campanhas de publicidade, relações públicas e canais de distribuição. O objetivo do *marketing* é entender e atender às necessidades do consumidor, criando valor para ambos, empresa e cliente. As estratégias de marketing são direcionadas para promover a marca da empresa, aumentar a sua visibilidade, atrair e reter clientes e, por fim, impulsionar as vendas de produtos ou de prestação de serviços e a lucratividade^(30)^.

O *marketing* pessoal, por outro lado, é a aplicação dos princípios do *marketing* para promover a marca de uma pessoa. É a definição e adoção de estratégias assertivas para aproximação da marca pessoal com o seu público-alvo, a fim de potencializar a sua divulgação, visibilidade e conexão em detrimento de novas oportunidades, colaborações, avanços na carreira e objetivos profissionais. Apresenta-se como uma ferramenta no cenário contemporâneo, onde se tornou ainda mais importante a autopromoção do indivíduo enquanto um profissional diferenciado, que agrega valor ao seu mercado de atuação^(8)^.

A partir das conceituações acima, é possível compreender que o *branding*, o *personal branding* e o *marketing* pessoal, apesar de distintos, podem funcionar em harmonia para consolidar um posicionamento robusto e diferenciado, seja de um negócio ou de um profissional, sendo que cada um desses elementos traz uma dimensão diferente para construção da identidade, reconhecimento e valorização de uma marca ou profissional. Logo, o *branding* e o *personal branding*, juntos, formam uma base sólida sobre a qual o *marketing* e o *marketing* pessoal podem atuar, comunicando e promovendo de maneira eficaz a identidade singular ao público-alvo.

Dos 16 estudos desta revisão, sete adotaram a expressão “*personal branding*” para se referir à marca pessoal de profissionais, o que demonstra uma diversidade terminológica e possivelmente de abordagens teóricas no cenário da enfermagem^(14,15,17-20,24)^. Essa variação pode indicar diferenças nas tradições acadêmicas ou nos contextos culturais em que os estudos foram conduzidos, bem como distintas interpretações sobre a construção e gestão da marca pessoal na enfermagem. Além disso, a escolha terminológica sugere que o conceito de marca pessoal ainda está em desenvolvimento, visto que são interpretados e aplicados de maneiras distintas pelos pesquisadores, evidenciando a riqueza e a complexidade do tema, seja para discussões ou para a sua aplicabilidade prática.

Também foi possível identificar que dois estudos utilizaram a expressão “*brand image*” (imagem da marca) ao abordar a marca pessoal do profissional^(26,27)^. É importante destacar que, embora “*brand image*” e “marca pessoal” estejam interligados, eles não são sinônimos, pois “*brand image*” se refere à percepção geral que os outros têm do profissional, enquanto “marca pessoal” envolve a construção consciente da identidade profissional. Segundo Barbosa^(31)^, a “*brand image*” é uma construção multifacetada, sustentada por três pilares integrados: comunicação verbal e não verbal, comportamentos, e aparência. No contexto da enfermagem, isso significa como o profissional é percebido com base em atributos como vocabulário (comunicação verbal), gestos, e expressões (comunicação não verbal) e atuação profissional.

A reflexão sobre essa distinção é crucial para entender como a imagem e a marca pessoal se diferenciam, mas podem se complementar. A “*brand image*” é, em grande parte, o resultado da marca pessoal bem gerida; é a maneira como o público percebe o profissional com base na narrativa pessoal que ele construiu. No entanto, a marca pessoal é mais proativa e abrangente, incluindo os esforços contínuos do profissional de enfermagem para desenvolver e comunicar uma identidade profissional coesa e autêntica. Sobretudo, em cenários de desigualdades sociais e condições precárias de trabalho, o branding poderia ser reconhecido como estratégia fundamentada em políticas públicas para aporte de visibilidade aos profissionais^(32,33)^.

O uso equivocado desses termos pode levar a uma compreensão superficial e a estratégias ineficazes para avanços da enfermagem no mercado de trabalho, podendo, até mesmo, promover compreensões inadequadas da identidade profissional. Portanto, é essencial que os estudos e práticas no campo da enfermagem reconheçam e respeitem essas nuances, garantindo que a construção da marca pessoal seja abordada de maneira holística e intencional. Ainda, nove artigos adotaram exclusivamente os termos “*brand*” e “*branding*” para discutir a marca pessoal na enfermagem, sem mencionar o termo “*personal*”^(12,16,19,22,25,27)^. Esse uso de termos mais gerais pode indicar uma tendência de aplicar conceitos de *branding* corporativo diretamente ao contexto individual dos profissionais de enfermagem. Embora “*brand*” e “*branding*” sejam conceitos essenciais para criação de uma identidade reconhecível, sua aplicação direta, sem considerar as particularidades próprias de personalização, pode levar a uma abordagem mais impessoal e genérica, potencialmente negligenciando a necessidade de autenticidade e individualidade, o que é essencial para a marca pessoal^(29)^.

Dos sete estudos que abordaram a temática *marketing*, cinco apresentaram o *marketing* em uma perspectiva mais ampla^(12,13,16,21,22)^, enquanto dois se concentraram na interface do *marketing* pessoal^(15,23)^. No entanto, uma reflexão analítica revela duas lacunas importantes. A primeira lacuna está relacionada aos seis artigos que trabalharam o *marketing* em uma perspectiva mais ampla, sem destacar as premissas do *marketing* pessoal, representando um desafio significativo ao serem adotados como referência para construção da marca pessoal para profissionais de enfermagem, pois enquanto o *marketing* tradicional pode abordar estratégias genéricas de promoção e visibilidade, o *marketing* pessoal vai além, enfatizando a identidade e os valores do indivíduo. Negligenciar essa dimensão pessoal pode resultar em uma abordagem imediata, superficial e ineficaz, que não captura a singularidade e autenticidade necessárias para o estabelecimento de uma reputação reconhecível do profissional, impactando diretamente o seu posicionamento e conexão com o público-alvo^(8)^. Já a segunda lacuna diz respeito aos dois estudos que trataram o *marketing* pessoal de maneira isolada, sem integrar a marca pessoal profissional nas discussões. Essa abordagem fragmentada ignora a complexa inter-relação entre *marketing* pessoal e marca pessoal, em que a última fornece a base essencial sobre a qual o *marketing* pessoal deve ser construído. Sem essa conexão, as estratégias de *marketing* pessoal podem se tornar insuficientes, focando em aspectos que não contribuam para uma reputação profissional duradoura, consistente e sustentável na enfermagem^(8)^.

Nessa perspectiva, o reconhecimento dos conceitos adotados se torna fundamental para que a enfermagem se auto-reconheça no real lugar de importância que ocupa no mercado de trabalho. O auto-(re)conhecimento desse processo pode contribuir, entre outros modos de constituição identitária, para o desenvolvimento de uma identidade profissional relevante e sustentável a longo prazo. Além disso, a personalização facilita a identificação de oportunidades específicas e a adaptação a diferentes contextos de trabalho, maximizando o impacto e a eficácia das estratégias de *personal branding* e *marketing* pessoal na carreira em enfermagem.

### Limitações do estudo

Consideram-se importantes limitações desta revisão a ausência da estratégia de busca em referências cruzadas e a não aplicação de critérios que avaliem a qualidade metodológica dos estudos. Além disso, a quantidade limitada de estudos incluídos e a escassez de pesquisas diretamente voltadas à enfermagem podem ter restringido a abrangência do mapeamento. É também possível que vieses interpretativos acerca dos conceitos tenham influenciado a delimitação do estudo.

### Contribuições para área da enfermagem

Provocar discussões acerca do (re)conhecimento dos conceitos de *branding, personal branding* e *marketing* pessoal no mercado de trabalho, desde a formação profissional, representa preencher uma lacuna no pensar e fazer da enfermagem para cultivar competências profissionais que transcendam o domínio técnico e clínico. Ao explorar esses conceitos na atuação desses profissionais, pode-se contribuir para dirimir desafios impostos em ambientes laborais por suas más condições para visibilizar o caráter empreendedor e de liderança da enfermagem.

## CONSIDERAÇÕES FINAIS

Os resultados desta revisão indicaram que, em nível global, ainda não há uma clara e estabelecida estrutura conceitual sobre *personal branding* e *marketing* pessoal na enfermagem. A ausência de uma base teórica consolidada nesse campo aponta para necessidade de futuros estudos que estabeleçam definições mais coesas, científicas e alinhadas com o contexto profissional e de mercado de trabalho para a enfermagem. Essas definições devem substituir ou complementar as abordagens fragmentadas atualmente disponíveis sobre *personal branding* e *marketing* pessoal na enfermagem. Ao propor conceitos mais alinhados com as demandas contemporâneas da profissão, espera-se estabelecer um fundamento teórico e prático que possa orientar os profissionais na promoção de suas marcas pessoais e nas decisões sobre as estratégias de *marketing* pessoal mais adequadas. Dessa forma, busca-se promover e fortalecer a identidade profissional.

Logo, é fundamental ressaltar que, embora a maioria dos estudos examinados tenha se concentrado no *branding* e *marketing,* em vez do *personal branding* e *marketing* pessoal ao explorar a marca pessoal na enfermagem, o entendimento desses conceitos também é igualmente crucial para os profissionais ocuparem os espaços que desejarem no mercado de trabalho em variados contextos.

## Data Availability

Os dados de pesquisa só estão disponíveis mediante solicitação.
